# Comparative Analysis of Ca^2^^+^/Cation Antiporter Gene Family in *Rosa roxburghii* and Enhanced Calcium Stress Tolerance via Heterologous Expression of RrCAX1a in Tobacco

**DOI:** 10.3390/plants13243582

**Published:** 2024-12-22

**Authors:** Tuo Zeng, Liyong Zhu, Wenwen Su, Lei Gu, Hongcheng Wang, Xuye Du, Bin Zhu, Caiyun Wang, Di Wu

**Affiliations:** 1 Guizhou Key Laboratory of Forest Cultivation in Plateau Mountain, School of Life Sciences, Guizhou Normal University, Guiyang 550025, China; zengtuo@gznu.edu.cn (T.Z.); leigu1216@nwafu.edu.cn (L.G.); wanghc@gznu.edu.cn (H.W.); duxuye@gznu.edu.cn (X.D.); 201703008@gznu.edu.cn (B.Z.); 2 National Key Laboratory for Germplasm Innovation & Utilization of Horticultural Crops, College of Horticulture & Forestry Sciences, Huazhong Agricultural University, Wuhan 430070, China; zly123456@webmail.hzau.edu.cn (L.Z.); wangcy@mail.hzau.edu.cn (C.W.); 3 Guizhou Institute of Mountain Resources, Guiyang 550025, China; 15591822196@163.com

**Keywords:** *Rosa roxburghii*, *CaCA* genes, Ca^2+^, metal stress

## Abstract

*Rosa roxburghii*, a calciphilic species native to the mountainous regions of Southwest China, is renowned for its high vitamin C and bioactive components, making it valuable for culinary and medicinal uses. This species exhibits remarkable tolerance to the high-calcium conditions typical of karst terrains. However, the underlying mechanisms of this calcium resilience remain unclear. The Ca^2^^+^/cation antiporter (CaCA) superfamily plays a vital role in the transport of Ca^2^^+^ and other cations and is crucial for plant tolerance to metal stress. However, the roles and evolutionary significance of the CaCA superfamily members in *R. roxburghii* remain poorly understood. This study identified 22 CaCA superfamily genes in *R. roxburghii*, categorized into four subfamilies. The gene structures of these *RrCaCAs* show considerable conservation across related species. Selection pressure analysis revealed that all *RrCaCAs* are subject to purifying selection. The promoter regions of these genes contain numerous hormone-responsive and stress-related elements. qRT-PCR analyses demonstrated that H^+^/cation exchanger (CAX) *RrCAX1a* and *RrCAX3a* were highly responsive to Ca^2^^+^ stress, cation/Ca^2^^+^ exchanger (CCX) *RrCCX4* to Mg^2^^+^ stress, and *RrCCX11a* to Na^+^ stress. Subcellular localization indicated that *RrCAX1a* is localized to the plant cell membrane, and its stable transformation in tobacco confirmed its ability to confer enhanced resistance to heavy Ca^2^^+^ stresses, highlighting its crucial role in the high-calcium tolerance mechanisms of *R. roxburghii*. This research establishes a foundation for further molecular-level functional analyses of the adaptation mechanisms of *R. roxburghii* to high-calcium environments.

## 1. Introduction

Calcium (Ca^2^^+^) is an essential element in plants, acting as a second messenger in response to extracellular signals. The Ca^2^^+^/cation antiporter (CaCA) superfamily is critical in regulating and accumulating calcium [1,2,3]. This superfamily encompasses distinct clades with varied phylogenetic, structural, and functional characteristics [4]. In plants, CaCA proteins are classified into four families: H^+^/cation exchangers (CAXs); Na^+^/Ca^2^^+^ exchanger-like proteins (NCLs); cation/Ca^2^^+^ exchangers (CCXs); and Mg^2^^+^/H^+^ exchangers (MHXs) [5,6]. Among these, CAXs and NCLs are implicated in abiotic stress responses [3,7], and play roles in hormone signaling and flowering [8,9]. CAXs may form heteromeric transporters influencing guard cell and mesophyll cell functions related to environmental adaptability [10]. Additionally, CaCA proteins are being explored for their potential in biofortification and phytoremediation due to their roles in metal ion uptake [11,12,13]. Notably, *CAX1a* in *Brassica rapa* and the heterologous expression of *Triticum urartu TuCAX1a* and *TuCAX1b* in *Arabidopsis thaliana*, play a pivotal role in calcium homeostasis, nutrient translocation, and metabolic regulation under calcium stress and heavy metal conditions [14,15]. *CAX1* also inhibits the formation of reactive oxygen species induced by Cd, a stress tolerance mechanism [16]. These varied reports highlight the multifunctionality of the CaCA gene family in plants.

*Rosa roxburghii*, commonly known as ‘Cili’ in China, is a wild deciduous perennial shrub of the Rosaceae family. It is noted for its golden, spine-covered fruits. The fruits emit a faint aroma and have a slightly tart taste but are primarily valued for their nutritional and medicinal benefits [17]. This shrub thrives in the cool, hilly regions of Southwestern China, particularly in Guizhou Province [18]. Beyond wild varieties, cultivated germplasm resources like ‘Guinong 5′ (Rr-5) were developed [19]. With growing research interest due to its medicinal and commercial values, *R. roxburghii* fruit is rich in carbohydrates, amino acids, vitamins, proteins, minerals, and dietary fibers. Other bioactive compounds, such as superoxide dismutase (SOD), organic acids, polysaccharides, flavonoids, polyphenols, triterpenes, and glycosides, contribute significantly to its medicinal properties. Studies have demonstrated its antioxidative, anti-tumor, anti-inflammatory, anti-radiation, anti-diabetic, and anti-aging effects [20,21,22]. *R. roxburghii* is also widely used in the food industry, enhancing the flavor of products such as herbal teas, jams, vinegar, yogurts, and mooncakes [23]. Its diverse applications and economic significance establish it as a valuable horticultural crop.

Originating from the karst regions of Southwestern China, *R. roxburghii* is a distinctive economic tree species with broad adaptability to various calcareous habitats [24]. As a calciphilic species, *R. roxburghii* demonstrates a robust response to the addition of exogenous Ca^2^^+^, marked by the upregulation of the genes involved in the ascorbate (AsA) synthesis pathway, including *L-galactono-1,4-lactone dehydrogenase* (*GalLDH*) and *galactose-1-phosphate phosphatase* (*GPP*), as well as genes related to AsA regeneration, such as *dehydroascorbate reductase* (*DHAR*) and *monodehydroascorbate reductase* (*MDHAR*) [25,26]. This leads to enhanced AsA synthesis and accumulation, which plays a pivotal role in improving the antioxidant capacity of *R. roxburghii* fruits. Exogenous Ca^2^^+^ also significantly promotes the accumulation of bioactive compounds, such as flavonoids and triterpenes, which contribute to the fruit nutritional and medicinal properties [27]. Despite multiple studies on the impact of calcium on the quality of *R. roxburghii*, comprehensive research on its adaptation to high-calcium conditions is still lacking. Therefore, this study was focused on the *RrCaCA* gene family, which plays a crucial role in heavy metal transport and stress responses, to explore the potential molecular mechanisms underlying *R. roxburghii’*s adaptation to high-calcium environments.

## 2. Result

### 2.1. Phylogenetic Analysis of RrCaCAs

**Figure 1:**
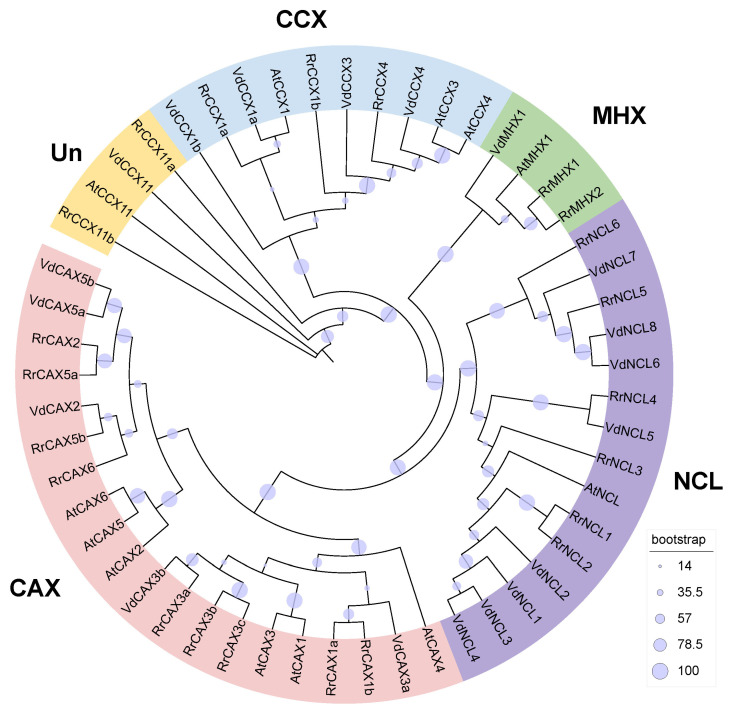
Phylogenetic tree of *CaCA* gene family. Constructed using the maximum likelihood (ML) method with 1000 bootstrap replications, the tree classifies 22 *CaCA* genes from *R. roxburghii*, 12 from *A. thaliana*, and 19 from *V. duclouxii* into 4 subfamilies.

Utilizing Hmmsearch combined with BLAST search based on *Arabidopsis CaCA* sequences, 22 *CaCA* genes were identified in *R. roxburghii*. Phylogenetic analysis revealed that these genes can be classified into four families: nine belong to the CAX family (*Rrox6G00416820*, *Rrox6G00416880*, *Rrox2G00097160*, *Rrox1G00011530*, *Rrox6G00416850*, *Rrox6G00416870*, *Rrox2G00097180*, *Rrox2G00093860*, *Rrox1G00006540*), six to the NCL family (*Rrox1G00048700*, *Rrox1G00048690*, *Rrox1G00048730*, *Rrox2G00115170*, *Rrox1G00048660*, *Rrox1G00066160*), five to the CCX family (*Rrox6G00389590*, *Rrox6G00389620*, *Rrox2G00146460*, *Rrox6G00425230*, *Rrox165G00437120*), and two to the MHX family (*Rrox7G00173680*, *Rrox7G00173670*). Based on their homologous genes in *Arabidopsis*, we further renamed these genes (Figure 1, Table S1).

The lengths of the RrCaCA protein range from 143 (RrCAX3c) to 655 (RrCCX4) amino acid residues. The molecular weight (MW) of these proteins ranges from 15,626.26 Da (RrCAX3c) to 71,192.28 Da (RrCCX4). The predicted isoelectric points (pI) of these proteins vary from 4.72 (RrCAX1b) to 9.43 (RrCCX11a). All proteins were acidic except RrCCX11a, and the instability index ranged from 26.00 (RrCAX3c) to 61.55 (RrCCX11a), with five proteins classified as unstable (instability index > 40) and the remainder considered stable. According to the grand average of hydropathy (GRAVY), RrCCX11a and RrCAX1b were hydrophobic, while the others were hydrophilic. RrCCX11b had the highest hydrophilicity index (0.81), and RrCCX11a had the lowest (−0.699). Subcellular localization predictions indicated that, except for RrCAX1b, which is predicted to be extracellular, and RrCCX11a, predicted to be nuclear, the remaining proteins are localized to the plasma membrane (Table S1).

### 2.2. Chromosomal Location and Collinearity Analysis of RrCaCAs

**Figure 2:**
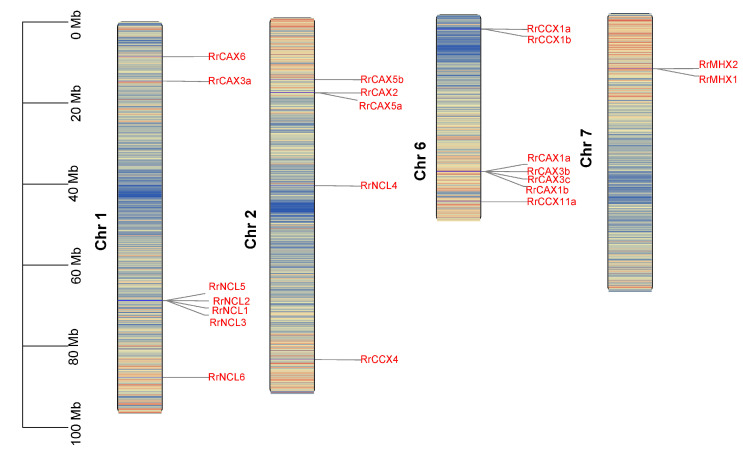
Chromosomal localization of *RrCaCA* genes. The chromosomal locations of the *RrCaCA* genes are depicted on Chr 1, Chr 2, Chr 6, and Chr 7. Genes are marked in red text to indicate their positions, with physical positions scaled by megabases (Mb) shown on the left. Chromosomal background shading represents gene density, where red indicates high gene density regions and blue indicates low gene density regions.

Genome annotation data reveal that the 22 *RrCaCA* genes are distributed across four of the seven chromosomes in *R. roxburghii*. Excluding *RrCCX11b*, which could not be mapped to any chromosome, the genes are primarily located on chromosomes 1 and 6, with each of these chromosomes hosting seven genes (Figure 2).

**Figure 3:**
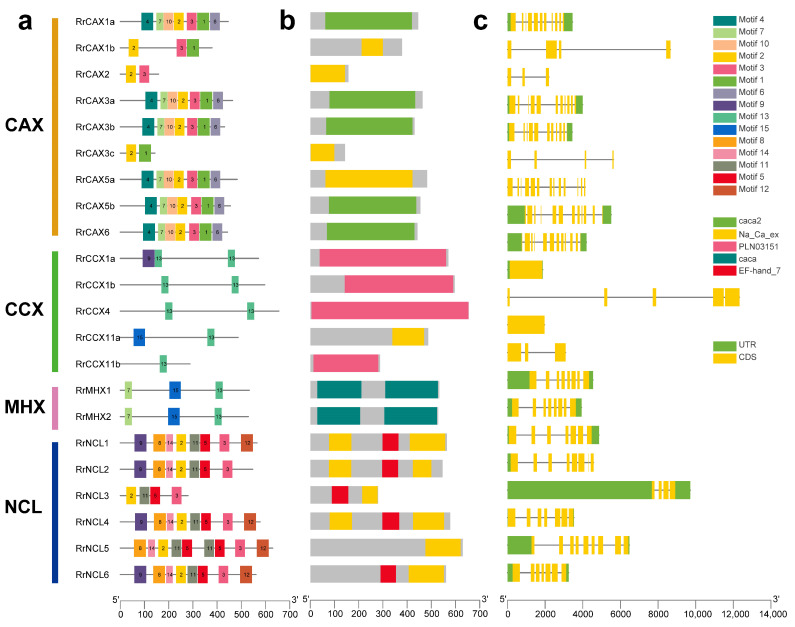
Structural motifs of *RrCaCA* genes. (**a**) MEME motifs; (**b**) conserved functional domains; (**c**) intron and exon structures.

The MEME software v5.5.7 was used to analyze 15 conserved motifs (Figure 3a). Motifs 2 and 3 were conserved across the *RrCaCA* genes and appeared in all subfamilies except CCX and MHX, indicating distinct motif compositions among subfamilies. The CAX subfamily contained motifs 2 and 3, with *RrCAX3c* lacking motif 3, and also included motifs 4, 15, 11, 12, 13, 8, and 14, suggesting a unique evolutionary pathway for this subfamily. The NCL subfamily primarily comprised motifs 2 and 3, along with unique motifs 5 and 11. In contrast, the CCX and MHX subfamilies contained motif 13 and exhibited unique motifs 13 and 7, pointing to a distinct evolutionary trajectory (Figure 3a). Conserved domain analysis showed that all *RrCaCA* genes contained at least one of the standard caca2, cacat, or Na_Ca_ex domains, with each gene having at least one of these conserved domains (Figure 3b).

The exon–intron structures of the 22 *RrCaCA* genes were analyzed (Figure 3c) based on intron number and exon length. The results revealed that structural diversity exists both within and between subclasses. While some members of the CAX subclass shared a relatively consistent exon–intron pattern, others exhibited significant variations in intron number and exon length. Similarly, the CCX subclass showed pronounced structural diversity among its members. The gain and loss of introns play a crucial role in altering gene architecture and are key to the evolutionary dynamics of gene families [28]. This indicated that the exon–intron structure within the subfamilies is relatively conserved, likely reflecting co-evolution with developmental processes specific to the *RrCaCA* family.

### 2.3. Duplication, Synthesis, and Evolutionary Analysis of RrCaCA Gene Family

**Figure 4:**
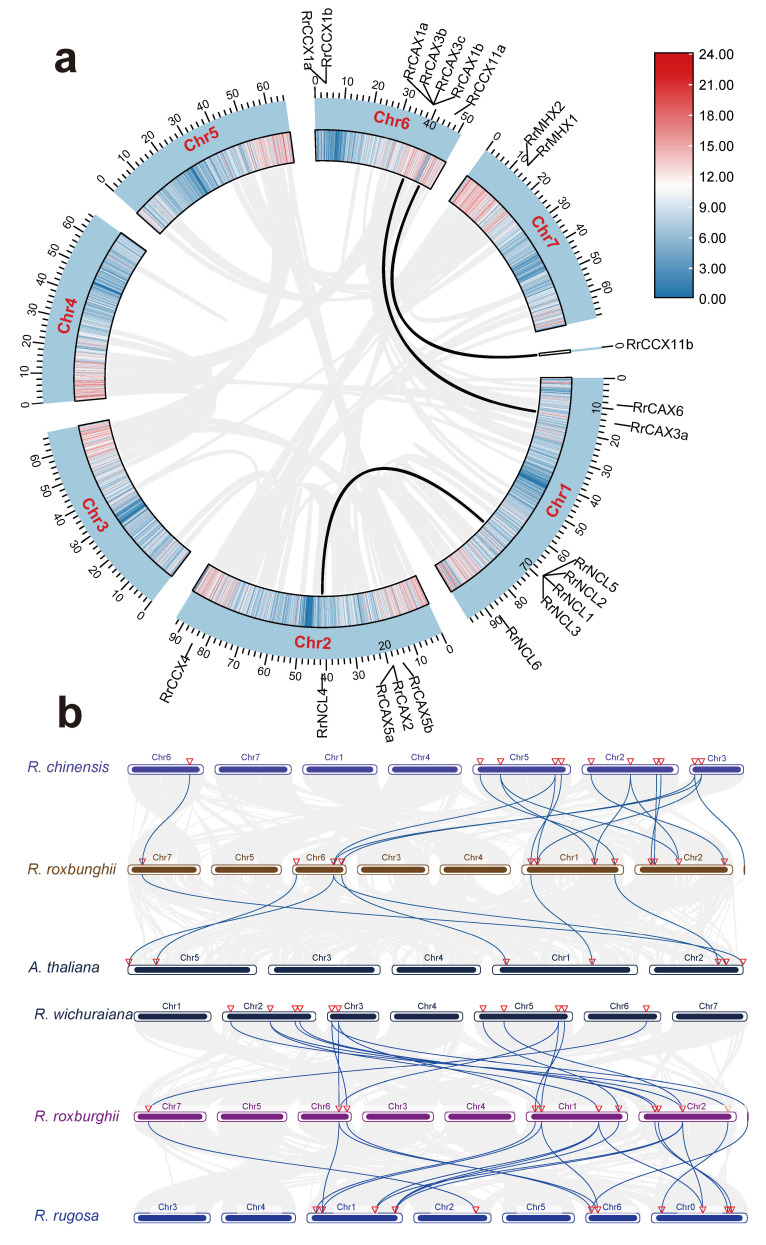
Collinearity analysis of *CaCA* genes. (**a**) Collinearity analysis of *RrCaCA* genes within *R. roxburghii* genome; (**b**) inter-genomic collinearity analysis among *A. thaliana*, *R. chinensis*, *R. wichuraiana*, and *R. rugosa*. Black and blue lines represent collinearity of *CaCA* genes.

Duplication events within the *RrCaCA* gene family were analyzed and visualized using MCScanX software v 1.098696 (Figure 4a). Among the 22 *RrCaCA* genes, 4 were categorized as tandem duplications, 3 as whole-genome duplications (WGD) or segmental duplications, 9 as proximal duplications, and 5 as dispersed duplications.

The evolutionary dynamics of the *RrCaCA* gene family were further elucidated through the identification of homologous genes selected from *A. thaliana*, *Rosa chinensis*, *Rosa wichuraiana*, and *Rosa rugosa* (Figure 4b). Six pairs of homologous gene pairs were identified between *R. roxburghii* and *A. thaliana,* eight between *R. roxburghii* and *R. wichuraiana*, and ten between *R. roxburghii* and *R. rugosa*, as well as between *R. roxburghii* and *R. chinensis*. The higher number of homologous pairs between *R. roxburghii* and other *Rosa* species, particularly *R. chinensis*, likely reflects their close phylogenetic relationships.

### 2.4. Analysis of Ka and Ks Substitution Patterns

**Figure 5:**
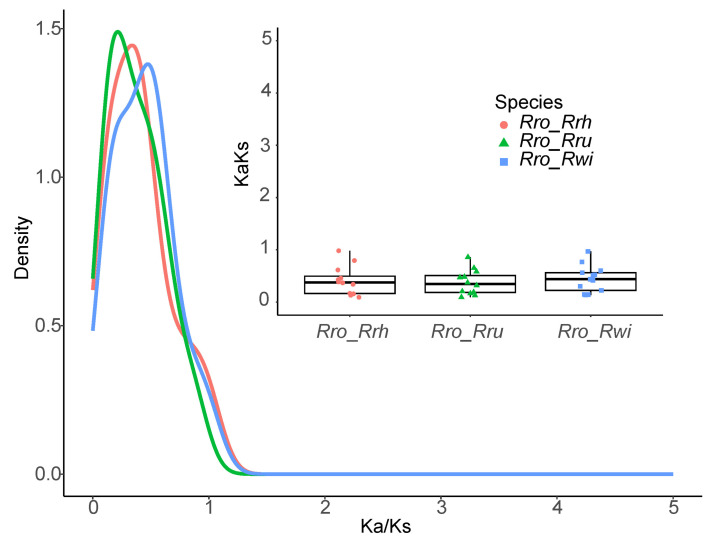
Ka/Ks values of *CaCAs* from four *Rosa* species: *R. roxburghii* (*Rro*), *R. chinensis* (*Rch*), *R. wichuraiana* (*Rwi*), and *R. rugosa* (*Rru*).

The patterns of synonymous (Ks) and nonsynonymous (Ka) nucleotide substitutions offer critical insights into the evolutionary mechanisms of genes. The Ka/Ks ratio, a fundamental metric in genetics, is employed to gauge the selective pressures on protein-coding genes and evaluate their diversification rates. A Ka/Ks ratio less than 1 signifies purifying selection, ratios greater than 1 indicate positive selection, and a ratio of 1 represents neutral selection [29]. To assess selective pressures, the Ka/Ks ratios for the *CaCA* gene family across four *Rosa* species were calculated. Genes with a Ka or Ks value of zero were excluded from the analysis. The average Ka/Ks ratio was 0.409, demonstrating that all genes examined were subject to purifying selection, with individual values ranging from 0.092 to 0.982. These results illustrate that the *RrCaCA* genes have experienced substantial purifying selection (Figure 5).

### 2.5. Identification of Cis-Acting Elements in Promoters of RrCaCAs

**Figure 6:**
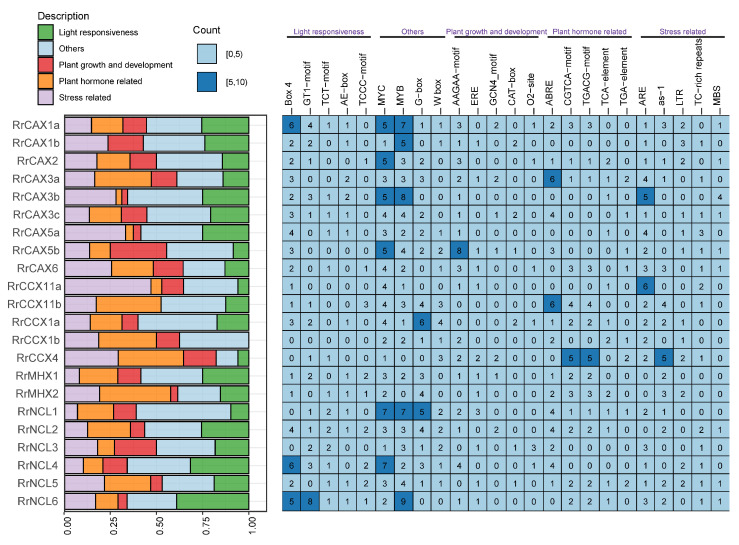
Heatmap showing predicted cis-acting elements in promoter regions of *RrCaCA*. The bar chart on the left visualizes promoter elements, with colors representing functional categories as indicated in the legend. The heatmap on the right shows the frequency of specific cis-regulatory motifs in the promoters of the analyzed genes, with numbers indicating motif occurrences.

The promoter regions of genes play a crucial role in transcriptional regulation. For the *RrCaCA* gene family, cis-acting regulatory elements were predicted using the PlantCARE online platform [30] (Figure 6). These elements include MYC, MYB, and G-box, which are primarily involved in light response, as well as Box 4, AAGAA-motif, and GCN4-motif, crucial in plant growth regulation. Hormone-responsive elements such as ABRE, CGTCA-motif, TCA-element, and TGA-element, along with stress-responsive elements like ARE and as-1, were also identified.

### 2.6. qRT-PCR Analysis of RrCaCAs Response to Heavy Metal Stress

**Figure 7:**
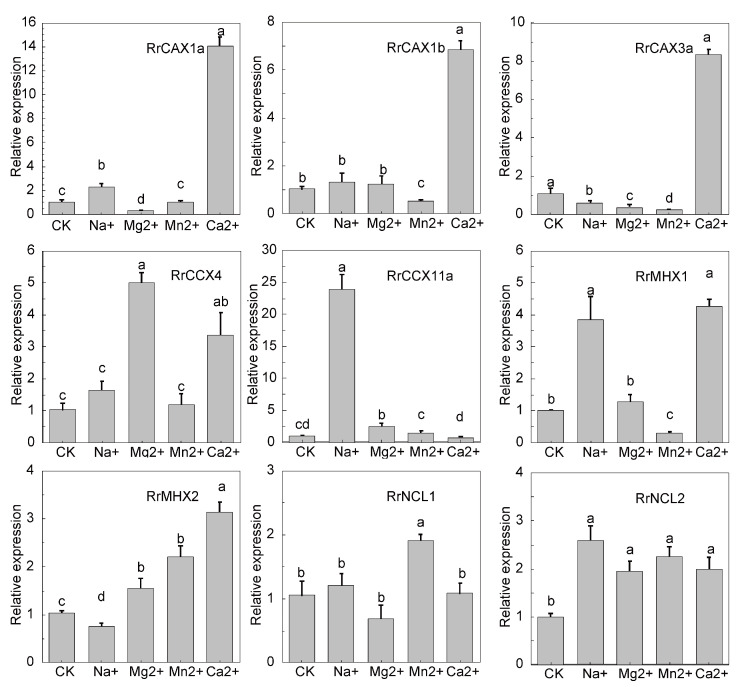
Expression profiles of *RrCaCA* under metal stress conditions. The qRT-PCR results normalized with the 2^−ΔΔCT^ Ct method using GADPH as an internal reference. The experiment was performed three times with three biological replicates. Error bars represent mean ± SD. CK refers to the untreated *R. roxburghii*, while the other treatments include *R. roxburghii* subjected to 200 mM NaCl, 50 mM MgCl_2_, 50 mM MnCl_2_, and 100 mM CaCl_2_. Different lowercase letters indicate significant differences, as determined by one-way ANOVA followed by Tukey’s post hoc test (*p* < 0.05). Groups with different letters are significantly different from each other, with the letter assignments based on the results of pairwise comparisons.

To further investigate the expression patterns of the *RrCaCA* genes in *R. roxburghii* under various heavy metal stresses, qRT-PCR analyses were conducted. The results indicated that *RrCAX1a* and *RrCAX1b* genes exhibited significant upregulation under Ca^2^^+^ treatment, with *RrCAX1a* reaching 14 times the control level and *CAX1b* about 8 times. Both genes showed reduced expression under Na^+^, Mg^2^^+^, and Mn^2^^+^ treatments. Similarly, *CAX3a* displayed a notable increase in expression under the Ca^2^^+^ treatment, approximately 8 times the control level, but decreased significantly under the Mn^2^^+^ treatment. *RrCCX4* and *RrCCX11a* also demonstrated the highest expressions under the Mg^2^^+^ and Ca^2^^+^ treatments, respectively, with *RrCCX11a* exceeding 20 times the control level. *RrMHX1* and *RrMHX2* showed the highest expressions under the Ca^2^^+^ treatment. In contrast, *RrNCL1* and *RrNCL2* exhibited minimal expression changes across all conditions (Figure 7). These findings underscore the significant role of the *RrCaCA* gene family in responding to calcium stress, highlighting their potential importance in heavy metal stress adaptation.

### 2.7. Subcellular Localization of RrCAX1a and Its Heterologous Expression Enhanced Tobacco Resistance to Calcium Stress

**Figure 8:**
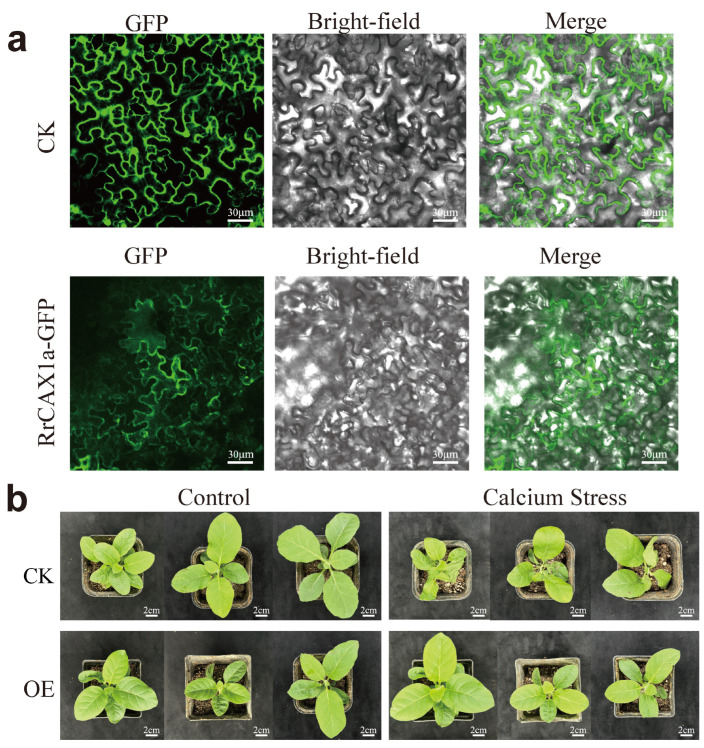
Subcellular localization of *RrCAX1a* and its role in enhancing calcium stress resistance in tobacco. (**a**) Subcellular localization of *RrCAX1a* showing its membrane-bound localization; CK represents control group transformed with empty pSuper1300-GFP vector. RrCAX1a-GFP indicates experimental group transformed with RrCAX1a-GFP fusion protein, demonstrating clear GFP signals localized to plasma membrane. GFP signals (**left**), bright-field images (**middle**), and merged images (**right**) are shown. Scale bar = 30 μm. (**b**) Overexpression of *RrCAX1a* in tobacco enhances resistance to calcium stress; CK represents transgenic tobacco plants transformed with pBI121 empty vector. OE represents transgenic tobacco plants transformed with pBI121-*RrCAX1a* vector. Scale bar = 2 cm.

To further verify the function of *RrCAX1a*, known for its high responsiveness to Ca^2^^+^ stress, we analyzed its subcellular localization. The coding sequence of *RrCAX1* was fused with green fluorescent protein (GFP). Transient expression in tobacco leaves revealed distinct green fluorescence on the cell membrane, confirming that *RrCAX1a* is localized to the cell membrane (Figure 8a). In *A. thaliana*, membrane-localized transporters such as *AtCAX1/3* are phosphorylated and activated by Ca^2^^+^ sensor kinase modules to facilitate Ca^2^^+^ efflux and maintain cytoplasmic homeostasis [31]. The localization of *RrCAX1a* to the cell membrane suggests its potential involvement in similar calcium transport mechanisms under Ca^2^^+^ stress conditions.

To investigate the function of *RrCAX1a* under Ca^2^^+^ stress, stable transformations were performed in tobacco using the pBI121 vector. Kanamycin resistance was used for selecting transformed plants. PCR amplification confirmed the successful integration of the *nptII* and *35S-RrCAX1a* sequences into the plant genome. Gel electrophoresis revealed clear bands of the expected sizes for these sequences, providing further validation of transgene integration (Figure S1). GUS staining also verified the transformation efficiency (Figure S2). The expression vector used in this study demonstrated high efficiency, resulting in the strong overexpression of *RrCAX1a* in transgenic lines. In contrast, the control (CK, transgenic tobacco plants transformed with the pBI121 empty vector) plants lacked detectable levels of *RrCAX1a* expression (Figure S3).

The phenotypic evaluation revealed that CK and *RrCAX1a*-overexpressing plants exhibited comparable growth under normal conditions. However, under Ca^2^^+^ stress, CK plants displayed severe symptoms, including wilting, leaf curling, and inhibited growth. In contrast, overexpression lines demonstrated significantly enhanced tolerance, maintaining healthier growth with minimal leaf curling (Figure 8b). These findings suggest that the overexpression of *RrCAX1a* enhances stress tolerance, potentially through the regulation of calcium ion homeostasis or interaction with stress response pathways. This provides valuable insights into breeding more stress-resistant plant varieties.

## 3. Discussion

*R. roxburghii*, a species within the genus *Rosa*, is predominantly found in the southwestern regions of China. Renowned for its remarkable resilience by thriving in nutrient-poor, rocky soils and high-calcium environments. Its capacity to grow under these challenging conditions makes it a valuable model for studying calcium tolerance. Understanding the mechanisms underlying this adaptability not only advances agricultural and ecological research but also provides potential strategies to enhance calcium tolerance in other crops, supporting sustainable agricultural practices in areas with suboptimal soil conditions.

### 3.1. RrCaCAs Structural Diversity and Evolutionary Insights

The structural diversity observed in the *RrCaCA* genes reflects both conservation and divergence within this family. Despite variations in intron–exon structure and motif composition (Figure 3), the conserved motifs (e.g., motifs 2 and 3) and domains (caca2, caca, and Na_Ca_ex) across most members indicate a core functional architecture. These findings suggest that variations in exon–intron structures and domain topologies are key evolutionary markers in gene families [32].

Gene duplication events, including tandem, proximal, and whole-genome duplications, have played a critical role in expanding the *RrCaCA* gene family (Figure 4). Such duplications increase gene counts and enable functional diversification [33,34]. The observed Ka/Ks ratios, averaging 0.409, indicate that most *RrCaCA* genes have undergone strong purifying selection, preserving their essential functions in calcium signaling and transport under evolutionary pressures.

### 3.2. RrCaCA Regulatory Elements and Stress Responses

Cis-acting elements identified in the promoters of *RrCaCA* genes, such as CGTCA-motif, TCA-element, ARE, and ABRE, reveal their roles in hormone signaling, stress responses, and growth regulation (Figure 6). These regulatory elements likely enable the fine-tuned expression of *RrCaCA* genes under diverse environmental conditions, contributing to the robust stress response of *R. roxburghii*. This aligns with previous reports highlighting the importance of promoter composition in facilitating plant responses to abiotic stress.

The expression analysis under metal stress further underscores the functional diversity of *RrCaCA* genes. For instance, *RrCAX1a* exhibited significant upregulation under calcium treatment, while other genes, such as *RrCCX4* and *RrCCX11a*, responded to magnesium stress (Figure 7). These differential expression patterns suggest that distinct members of the *RrCaCA* family are specialized for specific ion stress responses.

### 3.3. RrCaCA and RrCAX1a Mechanisms in R. roxburghii

Calcium transport in plants involves critical genes such as P2A- or P2B-type Ca^2^^+^-ATPases (ACAs) and CAXs, which actively remove excess Ca^2^^+^ from the cytoplasm to maintain ion homeostasis [35,36,37]. The knockout of *CAX1* has been shown to reduce cell wall extensibility [38]. Under NH_4_^+^ stress, calcium deficiency in tissues disrupts Ca^2^^+^ transport and localization in *Arabidopsis* leaves. The overexpression of *CAX1*, especially a modified version lacking its autoinhibitory domain, reduces apoplastic Ca^2^^+^ deficiency and alleviates NH_4_^+^-induced growth stunting by restoring Ca^2^^+^ homeostasis [39]. These findings suggest that *RrCAX1a*, identified in this study, likely plays a similar role in calcium transport and stress adaptation in *R. roxburghii*.

*CAX1* is the most highly expressed and differentially regulated transporter of Ca^2^^+^ between epidermal and mesophyll cells. The vacuolar localization of *CAX1* in Ca^2^^+^ transport was demonstrated through heterologous expression in yeast and other plant species [38]. Our functional validation of *RrCAX1a* supports this hypothesis. Subcellular localization analysis confirmed its plasma membrane localization. The overexpression of *RrCAX1a* in tobacco significantly enhanced calcium stress tolerance, as evidenced by reduced wilting and improved growth under high-calcium conditions (Figure 8). These results align with observations in tomato seedlings. The expression of *CAX1* correlates positively with Ca^2^^+^ accumulation, with the highest expression levels observed in leaves, whereas *CAX3* is more abundantly expressed in roots. In tomato seedlings treated with various ions, only *SlCAX1* and *SlCAX3* were significantly induced by the Ca^2^^+^ treatment, suggesting their roles in Ca^2^^+^ transport [40]. Similarly, *RrCAX1a* likely plays a pivotal role in *R. roxburghii* adaptation to high-calcium environments, contributing to its remarkable tolerance by regulating calcium homeostasis and mitigating calcium-induced stress.

### 3.4. Future Directions

The findings of this study provide valuable insights for developing stress-tolerant crops. The overexpression of *RrCAX1a* in tobacco demonstrates the potential of leveraging *RrCaCA* genes for enhancing calcium tolerance. Further studies are needed to elucidate the interactions between *RrCaCA* genes and other signaling pathways, such as those involving hormones and reactive oxygen species. The functional validation of additional *RrCaCA* members and their roles in other abiotic stress conditions will deepen our understanding of the molecular mechanisms underlying stress tolerance in *R. roxburghii*. Moreover, exploring the evolutionary dynamics of the *CaCA* gene family in other *Rosa* species can provide insights into the adaptive strategies of calcium tolerance across different environmental contexts.

## 4. Materials and Methods

### 4.1. Plant Materials

*R. roxburghii* seedlings were sourced from the Guizhou Institute of Mountain Resources and propagated via tissue culture. Seedlings of the *‘G*uinong No. *5′* variety, showing similar growth patterns, were selected and cultivated at the Huazhong Agricultural University Flower Research Base.

### 4.2. Identification and Phylogenetic Analysis of RrCaCA Gene Family in R. roxburghii

The *R. roxburghii* genome was sourced from NGDC (https://ngdc.cncb.ac.cn, accessed on 22 March 2024) [41]. Protein sequences for *A. thaliana* and *V. duclouxii* were retrieved from TAIR (https://www.arabidopsis.org/, accessed on 22 March 2024) and NGDC [42], respectively.

The identification of the *CaCA* superfamily, specifically those genes containing the Na_Ca_ex domain, was facilitated by the Pfam database (PF01699, http://pfam.xfam.org/, accessed on 24 March 2024) [43]. A search for Na_Ca_ex.hmm sequences was conducted using the HMMER v3.1b, targeting the protein databases of each species [44]. High-stringency BLAST searches (threshold of 1e-20) confirmed the *CaCA* candidates, which were further verified through domain checks in the NCBI Conserved Domain Database (CDD, https://www.ncbi.nlm.nih.gov/cdd, accessed on 25 March 2024). Physicochemical properties of the *RrCaCA*s were determined using the ExPASy-ProParam tool (https://web.expasy.org/protparam/, accessed on 28 March 2024). Predictions of subcellular localization were made using the CELLO prediction suite (http://cello.life.nctu.edu.tw/, accessed on 28 March 2024).

Phylogenetic relationships within the CaCAs of *R. roxburghii*, *A. thaliana*, and *V. duclouxii* were elucidated using MAFFT v7.158 for sequence alignment [45], and refined with the trimAL tool. Trees were constructed using the maximum likelihood method via iqtree2 with support from 1000 bootstrap replicates [46], and visualized with iTOL v5 [47].

### 4.3. Chromosomal Localization, Gene Structure Analysis, and Motif Identification of RrCaCAs

Using genomic annotation data from *R. roxburghii*, intron–exon structures were delineated to construct gene structure diagrams. Protein motifs were analyzed using the MEME Suite (https://meme-suite.org/meme/tools/meme, accessed on 28 March 2024). The chromosomal positions of the *RrCaCA* genes were pinpointed and illustrated using Tbtools (2024.1.11) [48].

### 4.4. Comparative Genomic Study of R. roxburghii and Other Rosa Species

Genomic sequences for *R. chinensis* [49], *R. rugosa* [50], and *R. wichuraiana* [51] were sourced from NCBI. The MCScanX was utilized to delineate syntenic relationships among the *RrCaCA* genes across these species. This comparative synteny was mapped and analyzed to explore evolutionary relationships. The Simple Ka/Ks Calculator in TBtools was used to determine Ka and Ks substitution rates for each gene pair, which helped assess the selective pressures they have undergone. These evolutionary insights were graphically represented using ggplot2 [52].

### 4.5. Analysis of Cis-Regulatory Elements in Promoters of RrCaCA Genes

Promoter regions, extending 2 kb upstream of the *RrCaCA* genes, were examined using the PlantCARE database (http://bioinformatics.psb.ugent.be/webtools/plantcare/html/, accessed on 15 September 2024) to identify potential cis-regulatory elements [30], and then visualized.

### 4.6. Stress Treatment of R. roxburghii

The stress treatment protocol for *R. roxburghii* was adapted from existing methods with slight modifications [40]. Thirty-day-old seedlings were exposed to various metal salt solutions, including 100 mM CaCl_2_, 50 mM MgCl_2_, 200 mM NaCl, and 50 mM MnCl_2_ for 16 h. All reagents were purchased from Macklin Biochemical Technology Co., Ltd. (Macklin, Shanghai, China), with a purity greater than 99%. For the control treatments, an equivalent volume of distilled water was applied. All plants were grown in a soil mixture of commercial substrate, leaf mold, and vermiculite in a 2:2:1 ratio. The plants were maintained under a 16 h light/8 h dark photoperiod, with a constant temperature of 25 ± 2 °C.

### 4.7. Validation of RrCaCAs with qRT-PCR

qRT-PCR analyses were performed to validate the expression of the *RrCaCAs*. RNA samples were reverse transcribed by a reverse transcription kit (Toyobo, Osaka, Japan), with specific primers checked by TBtools (Table S2). The qRT-PCR was carried out with the SYBR PreMix Ex Taq Kit (Takara, Kusatsu, Japan) on Roche LightCycler 96 System (Roche, Basel, Switzerland). The qRT-PCR protocol followed the parameters previously described [53], and gene expression levels were quantified using the 2^−ΔΔCT^ method [54], with GAPDH as the reference gene [55]. Each sample was subjected to three biological and two technical replicates to ensure the reliability of the results.

### 4.8. Subcellular Localization Analysis of RrCaCAX1a

The coding region of *RrCaCAX1a* without the stop codon was integrated into the *S*alI and *K*pnI linearized pSuper1300-GFP vector via ClonExpress^®^II One Step Cloning Kit (Vazyme, Nanjing, China). The constructed pSuper1300-*RrCaCAX1a*-GFP vector was transformed into *Agrobacterium tumefaciens* strain GV3101, and post-transformed to *Nicotiana benthamiana* as described [56]. After injection, the plants were incubated in darkness for 60 h. Green fluorescent protein (GFP) fluorescence was then examined using a confocal laser scanning microscope (Leica Microsystems TCS-SP8, Wetzlar, Germany).

### 4.9. The Genetic Transformation of Tobacco

The pBI121 vector was digested with *X*baI restriction enzyme, and the coding region of *RrCaCAX1a* without the stop codon was integrated into the vector via homologous recombination. The resulting recombinant vector was transformed into *A. tumefaciens* strain GV3101. Culture details and *Nicotiana tabacum* leaf disk transformation were performed as described [57]. The *Agrobacterium* was centrifuged at 5000× *g* for 5 min (USTC Zonkia Scientific Instruments, Heifei, China), resuspended in fresh YEB medium, and incubated for 1 h. The suspension was then centrifuged, resuspended in 1/2 MS medium (adjusted to an OD_600_ of 0.6), and used to infect tobacco leaf disks (approximately 0.8 × 0.8 cm) for 8 min. The leaf disks were then cultured in darkness at 25 °C for 3 days. After this period, the leaf disks were washed in 1/2 MS medium containing 400 mg/L Timentin for 5 min to remove residual *Agrobacterium*. They were then transferred to a selection medium containing kanamycin (100 mg/L) for plant regeneration. After 30 days, the regenerated shoots were transferred to the rooting medium. The well-rooted plants were subsequently acclimatized and grown under greenhouse conditions.

To confirm the successful integration of *RrCAX1a* into the tobacco genome, genomic DNA was extracted from transgenic plants, and PCR amplification was performed using primers specific for the nptII (kanamycin resistance) gene and *35S-RrCAX1a* (Table S2). Gel electrophoresis of the PCR products showed distinct bands corresponding to the expected sizes for these sequences, confirming the integration of the transgene. In addition, GUS staining was conducted following the instructions provided by the GUS reporter gene staining kit (Coolaber, Beijing, China) as described [58]. The transgenic tobacco plants were cultured in tissue culture bottles for seven days and then transferred to a 2:1 mixture of commercial substrate and vermiculite. After 20 days of growth at 25 ± 2 °C under a 16 h light/8 h dark cycle, the plants were treated with 100 mM CaCl_2_, and phenotypic changes were photographed 24 h later. The expression levels of *RrCAX1a* in overexpression lines were further quantified using qRT-PCR, with tobacco α-Tubulin as the internal reference gene [59].

## 5. Conclusions

This study effectively mapped and characterized the *R. roxburghii CaCA* gene family, identifying 22 genes distributed across 4 subfamilies. Despite high conservation within each subfamily, distinct sequence and structural variations across the subfamilies were noted. Promoter analyses revealed numerous hormone-responsive and stress-responsive elements, supporting the genes’ roles in metal stress response, particularly for *RrCAX1a*, which was shown to enhance calcium stress tolerance when expressed in tobacco. These findings lay the groundwork for further molecular studies on calcium tolerance in *R. roxburghii* and could inform future breeding programs aimed at enhancing the plant’s utility and resilience.

## Data Availability

Data are contained within the article and Supplementary Materials.

## Supplementary Materials

The following supporting information can be downloaded at: https://www.mdpi.com/article/10.3390/plants13243582/s1. Table S1: Prediction of physicochemical properties of *RrCaCAs*. Table S2: Primers used in experiment. Figure S1: PCR validation of transgene integration in transgenic tobacco plants. Figure S2: GUS staining confirmation of *RrCAX1a* transgene expression in tobacco leaves. Figure S3: Relative expression levels of RrCAX1a in transgenic tobacco lines.

## References

[1] Hirschi K.D., Zhen R.G., Cunningham K.W., Rea P.A., Fink G.R. (1996). CAX1, an H^+^/Ca^2+^ antiporter from *Arabidopsis*. Proc. Natl. Acad. Sci. USA.

[2] Morris J., Tian H., Park S., Sreevidya C.S., Ward J.M., Hirschi K.D. (2008). *AtCCX3* is an *Arabidopsis* endomembrane H^+^-dependent K^+^ transporter. Plant Physiol..

[3] Wang P., Li Z., Wei J., Zhao Z., Sun D., Cui S. (2012). A Na^+^/Ca^2+^ exchanger-like protein (AtNCL) involved in salt stress in *Arabidopsis*. J. Biol. Chem..

[4] Pittman J.K., Hirschi K.D. (2016). Phylogenetic analysis and protein structure modelling identifies distinct Ca^2+^/Cation antiporters and conservation of gene family structure within *Arabidopsis* and rice species. Rice.

[5] Taneja M., Tyagi S., Sharma S., Upadhyay S. (2016). Ca^2+^/Cation antiporters (CaCA): Identification, characterization and expression profiling in bread wheat (*Triticum aestivum* L). Front. Plant Sci..

[6] Amagaya K., Shibuya T., Nishiyama M., Kato K., Kanayama Y. (2019). Characterization and expression analysis of the Ca^2+^/cation antiporter gene family in tomatoes. Plants.

[7] Yamada N., Theerawitaya C., Chaum S., Kirdmanee C., Takabe T. (2014). Expression and functional analysis of putative vacuolar Ca2+-transporters (CAXs and ACAs) in roots of salt tolerant and sensitive rice cultivars. Protoplasma.

[8] Cho D., Villieres F., Kroniewicz L., Lee S., Seo Y.J., Hirschi K.D., Leonhardt N., Kwak J.M. (2012). Vacuolar *CAX1* and *CAX3* influence auxin transport in guard cells via regulation of apoplastic pH. Plant Physiol..

[9] Li P., Zhang G., Gonzales N., Guo Y., Hu H., Park S., Zhao J. (2016). Ca^2+^-regulated and diurnal rhythm-regulated Na^+^/Ca^2+^ exchanger AtNCL affects flowering time and auxin signalling in *Arabidopsis*. Plant Cell Environ..

[10] Hocking B., Conn S.J., Manohar M., Xu B., Athman A., Stancombe M.A., Webb A.R., Hirschi K.D., Gilliham M. (2017). Heterodimerization of *Arabidopsis* calcium/proton exchangers contributes to regulation of guard cell dynamics and plant defense responses. J. Exp. Bot..

[11] Morris J., Hawthorne K., Hotze T., Abrams S., Hirschi K.D. (2008). Nutritional impact of elevated calcium transport activity in carrots. Proc. Natl. Acad. Sci. USA.

[12] Navarro-León E., Ruiz J., Graham N., Blasco B. (2018). Physiological profile of CAX1a TILLING mutants of *Brassica rapa* exposed to different calcium doses. Plant Sci..

[13] Park S., Elless M.P., Park J., Jenkins A., Lim W., Edgar C.I.V., Hirschi K.D. (2009). Sensory analysis of calcium-biofortified lettuce. Plant Biotechnol. J..

[14] Navarro-León E., Ruiz J.M., Albacete A., Blasco B. (2019). Effect of *CAX1a* TILLING mutations and calcium concentration on some primary metabolism processes in *Brassica rapa* plants. J. Plant Physiol..

[15] Qiao K., Wang F., Liang S., Hu Z., Chai T. (2019). Heterologous expression of *TuCAX1a* and *TuCAX1b* enhances Ca^2+^ and Zn^2+^ translocation in *Arabidopsis*. Plant Cell Rep..

[16] Ahmadi H., Corso M., Weber M., Verbruggen N., Clemens S. (2018). *CAX1* suppresses Cd-induced generation of reactive oxygen species in *Arabidopsis halleri*. Plant Cell Environ..

[17] Su J., Zhang B., Fu X., Huang Q., Li C., Liu G., Hai Liu R. (2022). Recent advances in poly- saccharides from *Rose roxburghii* Tratt fruits: Isolation, structural character- ization, and bioactivities. Food Funct..

[18] Wang L., Wei T., Zheng L., Jiang F., Ma W., Lu M., Wu X., An H. (2023). Recent advances on main active ingredients, pharmacological activities of *Rosa roxbughii* and its development and utilization. Foods.

[19] Jiang L., Lu M., Rao T., Liu Z., Wu X., An H. (2022). Comparative analysis of Fruit Metabo- lome using widely targeted Metabolomics reveals nutritional characteristics of different *Rosa roxburghii* genotypes. Foods.

[20] Van der Westhuizen F.H., van Rensburg C.S.J., Rautenbach G.S., Marnewick J.L., Loots D.T., Huysamen C., Louw R., Pretorius P.J., Erasmus E. (2008). In vitro antioxidant, antimutagenic and genoprotective activity of *Rosa roxburghii* fruit extract. Phytother. Res..

[21] Wang L., Zhang B., Xiao J., Huang Q., Li C., Fu X. (2018). Physicochemical, functional, and biological properties of water-soluble polysaccharides from *Rosa roxburghii* Tratt fruit. Food Chem..

[22] Wang L., Zhang P., Li C., Xu F., Chen J. (2022). A polysaccharide from *Rosa roxburghii* Tratt fruit attenuates high-fat diet-induced intestinal barrier dysfunction and inflammation in mice by modulating the gut microbiota. Food Funct..

[23] Wang L.T., Lv M.J., An J.Y., Fan X.H., Dong M.Z., Zhang S.D., Wang J.D., Wang Y.Q., Cai Z.H., Fu Y.J. (2021). Botanical characteristics, phytochemistry and related biological activities of *Rosa roxburghii* Tratt fruit, and its potential use in functional foods: A review. Food Funct..

[24] Meng Q.-J., Fan W.-G. (2022). Calcium-tolerance type and adaptability to high-calcium habitats of *Rosa roxburghii*. Chin. J. Plant Ecol..

[25] Li L.L., An H.M. (2016). Effects of Ca^2+^ and Cu^2+^ on the expression of genes related to AsA metabolism in *Rosa roxburghii*. Fruits. J. Hortic..

[26] Zhang X., Yang M., An H.M., Huang W., Liu W. (2012). Effects of exogenous divalent cations Ca^2+^, Mg^2+^, and Cu^2+^ and acriflavine on ascorbate biosynthesis in *Rosa roxburghii* fruits. Chin. Agric. Sci..

[27] Wang Z., Lu M., An H. (2024). Transcriptome analysis reveals candidate genes involved in calcium absorption of *Rosa roxburghii* plants and their effects on the bioactive substance accumulation in fruit. J. Soil. Sci. Plant Nutr..

[28] Xu G., Guo C., Shan H., Kong H. (2012). Divergence of duplicate genes in exon–intron structure. Proc. Natl. Acad. Sci. USA.

[29] Hurst L.D. (2002). The Ka/Ks ratio: Diagnosing the form of sequence evolution. TRENDS Genet..

[30] Lescot M., Dehais P., Thijs G., Marchal K., Moreau Y., Van de Peer Y., Rouze P., Rombauts S. (2002). PlantCARE, a database of plant cis-acting regulatory elements and a portal to tools for in silico analysis of promoter sequences. Nucleic Acids Res..

[31] Wang C., Tang R.-J., Kou S., Xu X., Lu Y., Rauscher K., Voelker A., Luan S. (2024). Mechanisms of calcium homeostasis orchestrate plant growth and immunity. Nature.

[32] Lv W., Zhu L., Tan L., Gu L., Wang H., Du X., Zhu B., Zeng T., Wang C. (2024). Genome-wide identification and analysis of the *GST* gene family in wild blueberry (*Vaccinium duclouxii*) and their impact on anthocyanin accumulation. Plants.

[33] Magadum S., Banerjee U., Murugan P., Gangapur D., Ravikesavan R. (2013). Gene duplication as a major force in evolution. J. Genet..

[34] Flagel L.E., Wendel J.F. (2009). Gene duplication and evolutionary novelty in plants. New Phytol..

[35] Taneja M., Upadhyay S.K. (2021). An introduction to the calcium transport elements in plants. Calcium Transport Elements in Plants.

[36] Frei dit Frey N., Mbengue M., Kwaaitaal M., Nitsch L., Altenbach D., Häweker H., Lozano-Duran R., Njo M.F., Beeckman T., Huettel B. (2012). Plasma membrane calcium ATPases are important components of receptor-mediated signaling in plant immune responses and development. Plant Physiol..

[37] Pittman J., Hirschi K. (2024). CAX control: Multiple roles of vacuolar cation/H+ exchangers in metal tolerance, mineral nutrition and environmental signalling. Plant Biol..

[38] Conn S.J., Gilliham M., Athman A., Schreiber A.W., Baumann U., Moller I., Cheng N.-H., Stancombe M.A., Hirschi K.D., Webb A.A. (2011). Cell-specific vacuolar calcium storage mediated by *CAX1* regulates apoplastic calcium concentration, gas exchange, and plant productivity in *Arabidopsis*. The Plant Cell.

[39] Wdowiak A., Kryzheuskaya K., Podgórska A., Paterczyk B., Zebrowski J., Archacki R., Szal B. (2024). Ammonium nutrition modifies cellular calcium distribution influencing ammonium-induced growth inhibition. J. Plant Physiol..

[40] Han B., Tai Y., Li S., Shi J., Wu X., Kakeshpour T., Weng J., Cheng X., Park S., Wu Q. (2022). Redefining the N-terminal regulatory region of the Ca^2+^/H^+^ antiporter *CAX1* in tomato. Front. Plant Sci..

[41] Yang J., Zhang J., Yan H., Yi X., Pan Q., Liu Y., Zhang M., Li J., Xiao Q. (2024). The chromosome-level genome and functional database accelerate research about biosynthesis of secondary metabolites in *Rosa roxburghii*. BMC Plant Biol..

[42] Zeng T., He Z.J., He J.F., Lv W., Huang S.X., Li J.W., Zhu L.Y., Wan S., Zhou W.F., Yang Z.S. (2023). The Telomere-to-telomere gap-free reference genome of wild blueberry (*Vaccinium duclouxii*) provides its high soluble sugar and anthocyanin accumulation. Hortic. Res..

[43] El-Gebali S., Mistry J., Bateman A., Eddy S.R., Luciani A., Potter S.C., Qureshi M., Richardson L.J., Salazar G.A., Smart A. (2018). The Pfam protein families database in 2019. Nucleic Acids Res.

[44] Potter S.C., Luciani A., Eddy S.R., Park Y., Lopez R., Finn R.D. (2018). HMMER web server: 2018 update. Nucleic Acids Res..

[45] Katoh K., Standley D.M. (2013). MAFFT multiple sequence alignment software version 7: Improvements in performance and usability. Mol. Biol. Evol..

[46] Minh B.Q., Schmidt H.A., Chernomor O., Schrempf D., Woodhams M.D., von Haeseler A., Lanfear R. (2020). IQ-TREE 2: New models and efficient methods for phylogenetic inference in the genomic era. Mol. Biol. Evol..

[47] Letunic I., Bork P. (2021). Interactive Tree Of Life (iTOL) v5: An online tool for phylogenetic tree display and annotation. Nucleic Acids Res..

[48] Chen C.J., Chen H., Zhang Y., Thomas H.R., Frank M.H., He Y.H., Xia R. (2020). TBtools: An integrative toolkit developed for interactive analyses of big biological data. Mol. Plant.

[49] Hibrand Saint-Oyant L., Ruttink T., Hamama L., Kirov I., Lakhwani D., Zhou N.-N., Bourke P., Daccord N., Leus L., Schulz D. (2018). A high-quality genome sequence of *Rosa chinensis* to elucidate ornamental traits. Nat. Plants.

[50] Chen F., Su L., Hu S., Xue J.-Y., Liu H., Liu G., Jiang Y., Du J., Qiao Y., Fan Y. (2021). A chromosome-level genome assembly of rugged rose (*Rosa rugosa*) provides insights into its evolution, ecology, and floral characteristics. Hortic. Res..

[51] Zhong M.-C., Jiang X.-D., Yang G.-Q., Cui W.-H., Suo Z.-Q., Wang W.-J., Sun Y.-B., Wang D., Cheng X.-C., Li X.-M. (2021). Rose without prickle: Genomic insights linked to moisture adaptation. Natl. Sci. Rev..

[52] Wickham H. (2011). ggplot2. Wiley Interdiscip. Rev. Comput. Stat..

[53] Zeng T., Li J.W., Xu Z.Z., Zhou L., Li J.J., Yu Q., Luo J., Chan Z.L., Jongsma M.A., Hu H. (2022). *TcMYC2* regulates pyrethrin biosynthesis in *Tanacetum cinerariifolium*. Hortic. Res..

[54] Livak K.J., Schmittgen T.D. (2001). Analysis of relative gene expression data using real-time quantitative PCR and the 2^−ΔΔCT^ method. Methods.

[55] Shen Y., Sun T., Pan Q., Anupol N., Chen H., Shi J., Liu F., Deqiang D., Wang C., Zhao J. (2019). Rr MYB 5-and Rr MYB 10-regulated flavonoid biosynthesis plays a pivotal role in feedback loop responding to wounding and oxidation in Rosa rugosa. Plant Biotechnol. J..

[56] Zeng T., Yu Q., Shang J., Xu Z., Zhou L., Li W., Li J., Hu H., Zhu L., Li J. (2023). *TcbHLH14* a jasmonate associated MYC2-like transcription factor positively regulates pyrethrin biosynthesis in *Tanacetum cinerariifolium*. Int. J. Mol. Sci..

[57] Li J.W., Xu Z.Z., Zeng T., Zhou L., Li J.J., Hu H., Luo J., Wang C.Y. (2022). Overexpression of *TcCHS* increases pyrethrin content when using a genotype-independent transformation system in pyrethrum (*Tanacetum cinerariifolium*). Plants.

[58] Li J.W., Zeng T., Xu Z.Z., Zhou L., Shi A.Q., Luo Y.Y., Zhu L.Y., Wang Y.Y., Luo J., Wang C.Y. (2023). *TcWRKY75* participates in pyrethrin biosynthesis by positively regulating the expression of *TcCHS*, *TcAOC*, and *TcGLIP* in *Tanacetum cinerariifolium*. Ind. Crops Prod..

[59] Ma L., Jia W., Duan Q., Du W., Li X., Cui G., Wang X., Wang J. (2023). Heterologous expression of platycodon grandiflorus *PgF3′5′H.* modifies flower color pigmentation in tobacco. Genes..

