# Supplementary material for: Comparative Analysis of Ca2+/Cation Antiporter Gene Family in Rosa roxburghii and Enhanced Calcium Stress Tolerance via Heterologous Expression of RrCAX1a in Tobacco
Source: Plants (Basel). 2024 Dec 22;13(24):3582. doi: 10.3390/plants13243582 (PMC11677073; doi:10.3390/plants13243582)
Supplement: Supplementary file 1 [file plants-13-03582-s001.zip › plants-3331684-supplementary.pdf]

Supplemental information

Table S1: Prediction of physicochemical properties of the *RrCaCAs*

List	Gene ID	ID	Number of amino acids	Molecular weight	Theoretical pI	Instability index	Aliphatic index	Grand average of hydropathicity (GRAVY)	Predicted subcellular location
1	Rrox_6G00416820	RrCAX1a	446	48071.23	5.93	36.18	120.78	0.601	PlasmaMembrane
2	Rrox_6G00416880	RrCAX1b	379	41124.75	4.72	27.97	82.48	-0.296	Extracellular
3	Rrox_2G00097160	RrCAX2	158	17709.48	5.22	60.75	116.08	0.384	PlasmaMembrane
4	Rrox_1G00011530	RrCAX3a	464	50600.76	5.03	36.75	115.39	0.465	PlasmaMembrane
5	Rrox_6G00416850	RrCAX3b	431	47212.04	5.11	35.42	120.05	0.564	PlasmaMembrane
6	Rrox_6G00416870	RrCAX3c	143	15626.26	6.82	26	113.29	0.416	PlasmaMembrane
7	Rrox_2G00097180	RrCAX5a	483	53179.25	5.21	36.14	115.84	0.567	PlasmaMembrane
8	Rrox_2G00093860	RrCAX5b	455	49349.94	5.38	34.45	115.93	0.563	PlasmaMembrane
9	Rrox_1G00006540	RrCAX6	443	48798.08	5.15	34.59	110.05	0.476	PlasmaMembrane
10	Rrox_6G00389590	RrCCX1a	571	61919.21	5.81	27.73	120.68	0.569	PlasmaMembrane
11	Rrox_6G00389620	RrCCX1b	597	64950.78	6.8	48.93	106.75	0.219	PlasmaMembrane
12	Rrox_2G00146460	RrCCX4	655	71192.28	6.61	34.14	110.6	0.559	PlasmaMembrane
13	Rrox_6G00425230	RrCCX11a	487	55879.93	9.43	61.55	80.74	-0.699	Nuclear
14	Rrox_165G00437120	RrCCX11b	288	31514.47	5.67	41.55	121.53	0.81	PlasmaMembrane
15	Rrox_7G00173680	RrMHX1	533	59051.75	5.37	36.29	114.15	0.515	PlasmaMembrane
16	Rrox_7G00173670	RrMHX2	529	58675.23	5.08	37.08	115.18	0.542	PlasmaMembrane

17	Rrox_1G00048700	RrNCL1	565	61832.92	5.45	30.31	117.66	0.41	PlasmaMembrane
18	Rrox_1G00048690	RrNCL2	547	59450.67	5.39	30.93	110.15	0.239	PlasmaMembrane
19	Rrox_1G00048730	RrNCL3	280	31444.37	5.53	40.81	110.36	0.14	PlasmaMembrane
20	Rrox_2G00115170	RrNCL4	578	63885.6	5.16	35.27	117.34	0.482	PlasmaMembrane
21	Rrox_1G00048660	RrNCL5	630	70877.67	5.07	38.81	109.51	0.193	PlasmaMembrane
22	Rrox_1G00066160	RrNCL6	561	61874.32	6.55	34.81	113.99	0.488	PlasmaMembrane

Table S2: Primers used in experiment

Experiments used	Primer name	Primer sequences（5' to 3'）
Realtime PCR	CAX1a-F	AGGAGGAATCGGATGATGATGA
Realtime PCR	CAX1a-R	GCCAACAATAGGTAGCAGGATTA
Realtime PCR	CAX1b-F	AGTCGCTGGTGCTGATGTT
Realtime PCR	CAX1b-R	GGTATGTACGCCACTGGAGTA
Realtime PCR	CAX3a-F	AGAATAAGGAGCAACGGAGTGT
Realtime PCR	CAX3a-R	GCGAGTGGAATGGCAGGAA
Realtime PCR	CCX11a-F	GACCTACAGCGACGATACCA
Realtime PCR	CCX11a-R	GCTTCTTCTTGCTCATCAGTGT
Realtime PCR	CCX4-F	TGTCTCTGCTTCCGCTTGG
Realtime PCR	CCX4-R	TCCTATCAATCTGAACCGCCTTA
Realtime PCR	GADPH-F	TGAAGGGTGGTGCCAAGAA
Realtime PCR	GADPH-R	AAGGGGAGCAAGACAGTTGG
Realtime PCR	MHX1-F	TTACCGCATTAGCAAGTGGAA
Realtime PCR	MHX1-R	CTCAATCCGCAGTGGTTCTT
Realtime PCR	MHX2-F	CCACTGCGGATTGAGAAT
Realtime PCR	MHX2-R	AGAGAAGACAGCACAACAA
Realtime PCR	NCL1-F	GAGGCTGTATCTGCGATAATCTT
Realtime PCR	NCL1-R	ACGATAACAATGACGAGGACTTC
Realtime PCR	NCL2-F	TCCTTGACGATAATGGTGAACTC
Realtime PCR	NCL2-R	CAGCATCATCCTTATCCAACTCTAT

Genetic transformation	CAX1a-pBI121-F	AGAACACGGGGGACTCTAGAATGGCTTCAAACCAAGAACC
Genetic transformation	CAX1a-pBI121-R	CCACCCGGGGATCCTCTAGAAGCTCCAAAGACTGCTCCAG
Genetic transformation	CAX1a-ps1300-F	TGCAGGGGCCCGGGGTCGACATGGCTTCAAACCAAGAACC
Genetic transformation	CAX1a-ps1300-R	CCCTTGCTCACCATGGTACCAGCTCCAAAGACTGCTCCAG
Detection primer	35s-CAX1-F	CCACTATCCTTCGCAAGACC
Detection primer	35s-CAX1-R	GGACCGGTGAAAAAAGCAATC
Detection primer	nptII-F	CTATTCGGCTATGACTGGGC
Detection primer	nptII-R	GCAGGAGCAAGGTGAGATGAC
Realtime PCR	α-Tubulin-F	ATGAGAGAGTGCATATCGAT
Realtime PCR	α-Tubulin-R	TTCACTGAAGAAGGTGTTGAA

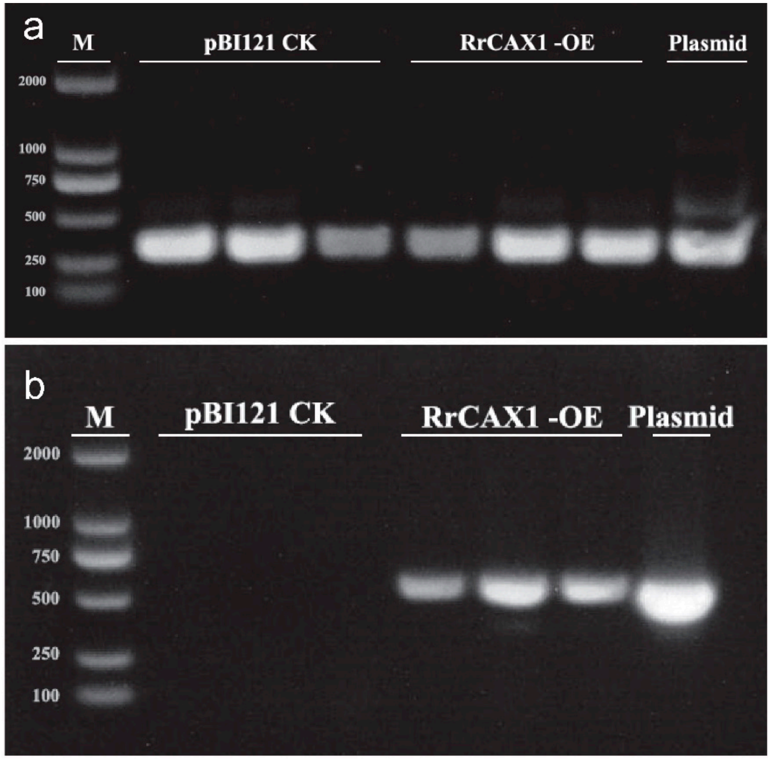

Figure S1. PCR validation of transgene integration in transgenic tobacco plants.

a: Amplification of the nptII gene fragment in genomic DNA extracted from transgenic tobacco plants. b: Amplification of the 35S-RrCAX1a fusion sequence in genomic DNA. **pBI121 CK** represents transgenic tobacco plants transformed with the pBI121 empty vector sequence, **RrCAX1a-OE** represents transgenic tobacco plants transformed with the pBI121-*RrCAX1a* Plasmid corresponds to the positive control using the original transformation plasmid, and **M** is the molecular weight marker.

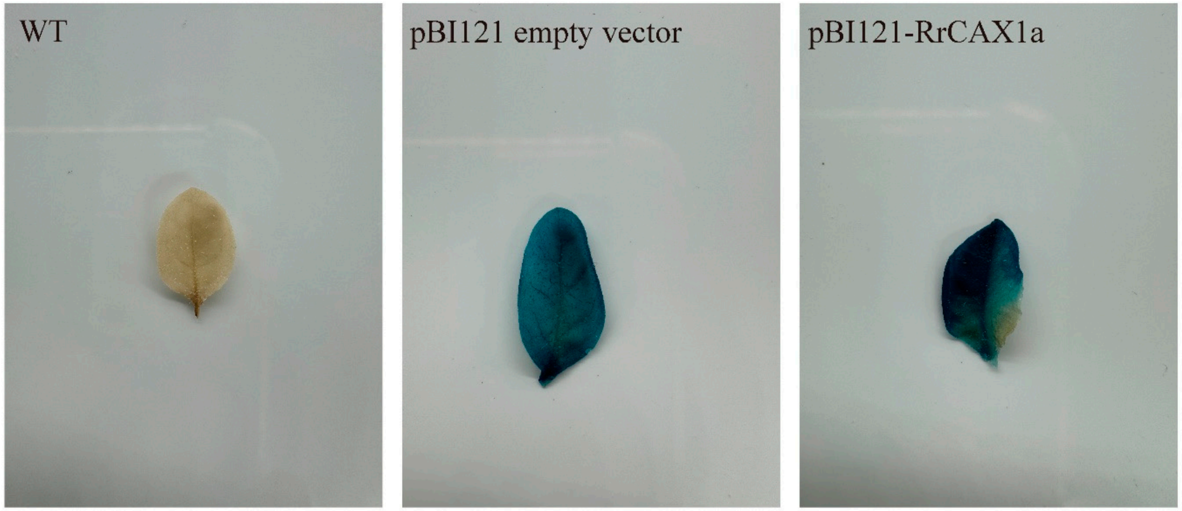

Figure S2 GUS staining confirmation of *RrCAX1a* transgene expression in tobacco leaves

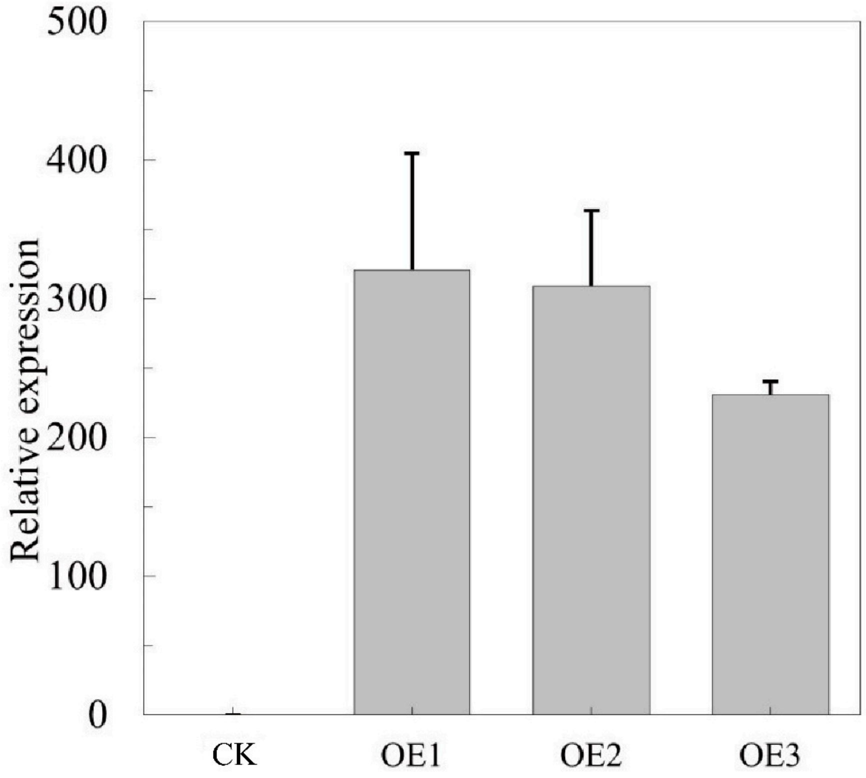

Figure S3. Relative expression levels of *RrCAX1a* in transgenic tobacco lines.

Quantitative PCR (qPCR) analysis of *RrCAX1a* expression in control (CK) and three overexpression lines (OE1, OE2, OE3) of transgenic tobacco. CK represents plants transformed with the pBI121 empty vector, showing negligible expression of *RrCAX1a*. In contrast, the overexpression lines (OE1–OE3) exhibit significantly elevated expression levels of *RrCAX1a*, with relative expression levels normalized to WT. Data are presented as the mean ± SD from three biological replicates.
